# Optimization Design and Flexible Detection Method of Wall-Climbing Robot System with Multiple Sensors Integration for Magnetic Particle Testing

**DOI:** 10.3390/s20164582

**Published:** 2020-08-15

**Authors:** Xiaojun Zhang, Xuan Zhang, Minglu Zhang, Lingyu Sun, Manhong Li

**Affiliations:** School of Mechanical Engineering, Hebei University of Technology, Tianjin 300130, China; xjzhang@hebut.edu.cn (X.Z.); 201811201016@stu.hebut.edu.cn (X.Z.); zhangml@hebut.edu.cn (M.Z.); sunly@hebut.edu.cn (L.S.)

**Keywords:** wall-climbing robot, multiple sensors integration, magnetic particle testing, flexible detection

## Abstract

Weld detection is vital to the quality of ship construction and navigation safety, and numerous detection robots have been developed and widely applied. Focusing on the current bottleneck of robot safety, efficiency, and intelligent detection, this paper developed a wall-climbing robot that integrates multiple sensors and uses fluorescent magnetic powder for nondestructive testing. We designed a moving mechanism that can safely move on a curved surface and a serial-parallel hybrid flexible detection mechanism that incorporates a force sensor to solve the robot’s safe adsorption and a flexible detection of the curved surface to complete the flaw detection operation. We optimized the system structure and improved the overall performance of the robot by establishing a unified mechanical model for different operating conditions. Based on the collected sensor information, a multi-degree of freedom component collaborative flexible detection method with a standard detecting process was developed to complete efficient, high-quality detection. Results showed that the developed wall-climbing robot can move safely and steadily on the complex facade and can complete the flaw detection of wall welds.

## 1. Introduction

Steel assembly welding is the main technology in hull construction; thus, numerous welds will be generated. During construction and maintenance phases, weld detection is an effective measure to ensure ship quality navigation safety [1,2]. The fluorescent magnetic particle testing technology can adapt to a complex, harsh environment, and quickly display the position and size of the defect shape [3,4,5], which is the most commonly nondestructive testing widely used in the ship maintenance [6]. Existing fluorescent magnetic particle testing methods are mainly carried out by workers handheld devices, which have problems of low efficiency, inability to save and manage data, and operation safety. Therefore, combining wall-climbing robot technology and the fluorescent magnetic particle testing technology, designing a detection robot that can replace manual operations has become the key to speeding up equipment replacement and process upgrade in the field of ship inspection and repair, and is a research hotspot in robot technology [7,8,9,10].

At present, extensive research has been carried out on mobile mechanisms and detection devices of ship detection robots; several typical robot systems have been developed and applied in several fields [11]. Zhang et al. [12] and Zhu et al. [13] carried out magnetic circuit optimization research and developed a new type of permanent magnet wheel with high magnet utilization by adjusting the structural parameters of the magnetic wheel. Their research is more suitable for application in large wall-climbing detection mechanism due to large external dimensions and weight of the wheel. Todd Adkins et al. [14] designed a device based on eddy current testing model to realize wall defect detection, but it is difficult to be applied to automatic engineering application temporarily. Dai et al. [15] and Yin et al. [16] successively carried out research on pipeline weld defect detection technology and developed robots for pipeline non-destructive testing, whose work efficiency can be improved in the following research. Several compound mobile mechanisms have been innovatively designed to improve the wall adaptability of a wall-climbing robot, but the conflict between movement flexibility and safe adsorption has not been well resolved [17,18,19]. Several researchers carried detection sensors and cleaning equipment on a wall-climbing robot and developed a series of ship detection and cleaning robots [20,21,22,23,24,25,26,27]. Due to the lack of flexibility in the connection between the end-effector and the body, extremely accurate controls are commonly applied to meet detection requirements, making the control more difficult. Jiang et al. [28] and Zhao et al. [29] proposed a mechanical analysis method that combines parameters such as load, adsorption force, and spatial angle, and carried out robot mechanical analysis to optimize the structure, which improved the comprehensive performance of the robot. Ryo et al. [30] and Dong et al. [31] researched the flexible motion control method of the wall-climbing robot to improve the robot’s compliant operation ability.

Existing detection robots still have shortcomings in terms of mobile mechanisms, detecting devices, system optimization, and technical process, mainly as follows: 1. The insufficient adsorption force of the moving mechanism makes it difficult for stable accurate movement on the curved surface in space. How to design a lightweight magnetic wheel with high adsorption force that can adapt to the arc wall surface is a difficult problem to be solved in this field. 2. Detection device is difficult to achieve compliant self-adaptation and complete accurate measurement of lifting force on walls with different curvature radii. For different wall environments, the design of an adaptive flexible detection mechanism and the accurate measurement method of lifting force without displacement are the keys to ensure the validity of detection results. 3. It is difficult to solve the contradiction between the robot’s weight and its adsorption force because of the lack of unitive mechanical analysis under different operating conditions, which affects the overall performance of the robot. Solving the contradiction between weight and adsorption force in the structure optimization through system modeling and analysis is an urgent problem. 4. Existing detection robots cannot achieve the close coordination and cooperation of multiple execution parts so that the traditional process is difficult to achieve efficient operations. Setting up sensors and using sensed information to realize efficient autonomous control of complex processes has been a trend of industrial automation.

This paper introduces the wall-climbing robot system used in the ship’s complex curved surface fluorescent magnetic particle testing. We propose an array-type permanent magnet adsorption wheel, which uses a mechanical structure to improve the effective utilization of magnetic force and the safe, stable movement of the robot on the space wall. We design a passive flexible adaptive detection mechanism based on serial-parallel hybrid elastic elements to achieve adaptive and accurate measurement of the detection equipment under different curvature radius surfaces. We establish a system mechanical model of the robot based on a time-varying force field and then optimize the system structure to improve the overall performance of the robot. We combine the model with multi-sensor fusion technology, deeply integrate the standard inspection workflow and the inspection robot’s multi-execution component actions, and then develop an automated process flow suitable for magnetic particle inspection.

The remainder of this paper is organized as follows. Section 2 introduces the structure of the detection robot, which mainly includes the magnetic adsorption moving mechanism and the passive flexible detection mechanism. Section 3 establishes a unified mechanical model based on time-varying force field parameters for system optimization. Section 4 introduces the hardware composition of the control system and the automatic and efficient detection of fusing multi-sensor information. Section 5 introduces the experimental process and analysis results. Section 6 provides several conclusions drawn from this research.

## 2. Introduction to Detection Robot

**Figure 1:**
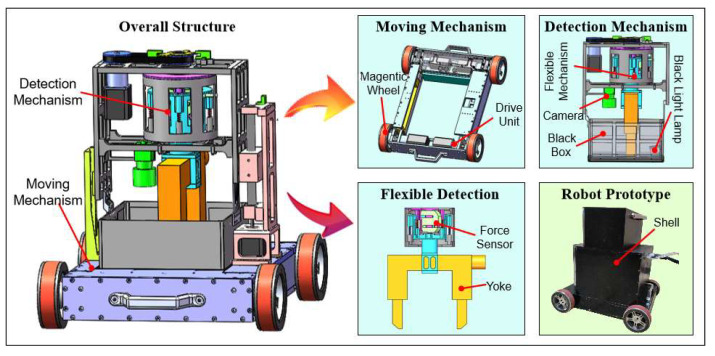
Overall architecture of the detection robot.

The detection robot designed in this paper is mainly composed of a four-wheel-drive moving mechanism with adsorption force and a passive adaptive detection mechanism. We need to solve the stable accurate movement of the moving mechanism, the self-adaptation of the curved surface of the detection mechanism, and the accurate measurement of lifting force because the robot mainly carries out the welding detection operation under the continuously variable curvature facade. In engineering applications, weld detection is usually carried out in an anhydrous plant, so we mainly design for such operating conditions. Therefore, a four-wheel-drive mobile body is designed, and a magnetic wheel with array-type permanent magnets is proposed to ensure reliable adsorption of the mobile mechanism and meet the requirements of robot motion accuracy. A serial-parallel hybrid flexible mechanism is designed to solve the compliance of different curved surfaces and make the detection equipment close to the wall surface. The force sensor in the flexible mechanism realizes the accurate measurement of the lifting force. Simultaneously, image acquisition, magnetic suspension spraying, and yoke rotary lifting mechanism are also included in the robot system. Finally, the robot’s multi-mechanisms are coordinated and controlled to complete the automated inspection through the control system and multi-sensors information fusion, such that the robot can perform standard efficient intelligent inspection operations under complex walls. The overall structure of the detection robot is shown in Figure 1:

### 2.1. Magnetic Wheel Design and Optimization

In the work of a facade with magnetic conductivity, the magnitude of a robot’s adsorption force affects directly the movement’s reliability; thus, the design and optimization of the adsorption device are keys to ensuring the movement’s reliability. The greater the adsorption force is, the better the robot movement reliability. However, the improvement of the adsorption force is mainly achieved by increasing the weight of the magnet, which leads to a technical conflict between the improvement of the adsorption force and its weight. Therefore, the design of a lightweight adsorption device is crucial. Different from other adsorption mechanisms, this paper uses pure iron with a high magnetic conductivity to collect magnetic lines of all permanent magnets, forms the shortest magnetic circuit with the wall surface, thus improving the utilization ratio of magnetic energy product, and obtains a permanent magnet wheel with lightweight strong adsorption force.

**Figure 2:**
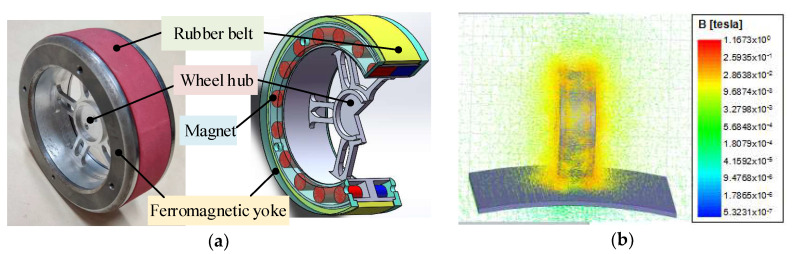
Magnetic wheel. (**a**) Schematic diagram and object; (**b**) magnetic simulation results.

The magnetic wheel consists of two ferromagnetic yokes, 20 cylindrical permanent magnets, a rubber belt, and a wheel hub made of an aluminum synchronous pulley, as shown in Figure 2. Permanent magnets are evenly installed in the wheel hub, both sides of which are ring-shaped yokes coaxial with the hub. A synchronous belt is wrapped around the outside of the hub in a meshing manner. The magnets produce magnetic flux through the ferromagnetic yokes and the ferromagnetic wall that result in a constant strong attractive force between them to keep the robot stay on the surface. The belt protects the wall from scratches and can increase the friction between wheel and wall to allow the robot to climb stably without slippage by using its deformation.

**Figure 3:**
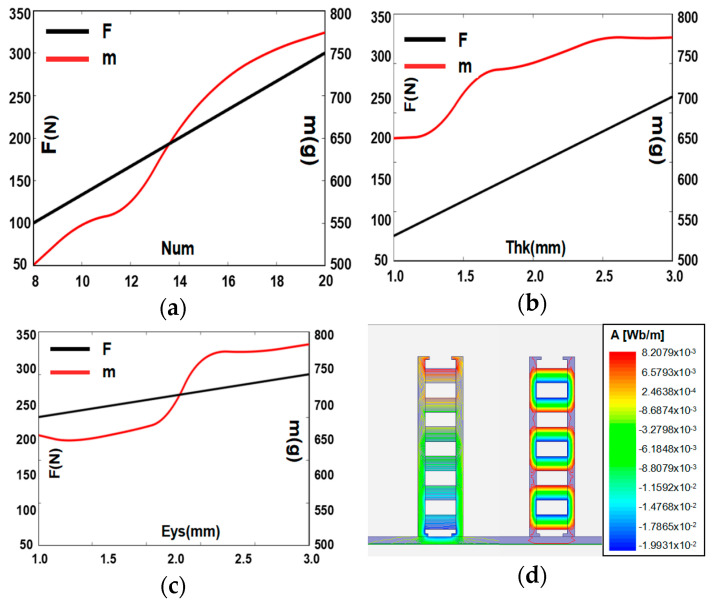
Influence of structural parameters on the magnetic wheel. (**a**) Number of magnets (Num); (**b**) thickness of the yoke (Thk); (**c**) thickness of the yoke hole shoulder (Eys); (**d**) arrangement.

Aiming at the above structure, the simulation results on the adsorbability of the magnetic wheel are carried out. We compare the results and optimize the structural parameters of the wheel to obtain a lightweight wheel with strong adsorption. We find through simulation that the magnet’s performance (number and arrangement) and the yoke performance (the thickness and the thickness of the whole shoulder) in the magnetic wheel structure are directly related to the adsorption force. As shown in Figure 3a, as the number of magnets increases, the magnetic flux density of the magnetic wheel increases, which greatly improves the adsorption force of the magnetic wheel. As shown in Figure 3b, as the thickness of the yoke increases, the yoke’s ability to collect and conduct magnetic induction lines is enhanced, which increases the strength of the magnetic field on the contact wall and improves the magnetic wheel’s suction force. As shown in Figure 3c, as the thickness of the yoke hole shoulder increases, the contact area between the magnetic wheel and the surface is enlarged, which improves the magnetic circuit transmission efficiency, and then improves the adsorption force of the magnetic wheel. As shown in Figure 3d, comparing the two different arrangements, a strong, dense magnetic induction loop can be formed when the magnets are arranged in the same polarity. Moreover, the adjacent magnet has no loop to cause the effective magnetic field strength to decrease. Therefore, the magnetic wheels in which the magnets are arranged in the same polarity can obtain a higher adsorption force.

**Figure 4:**
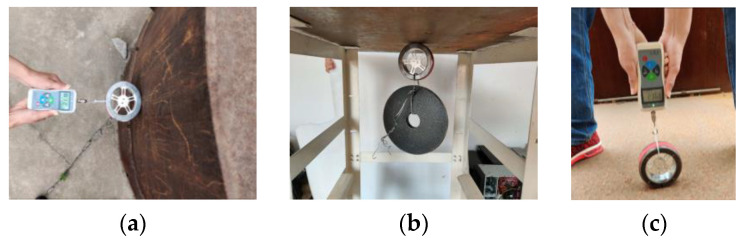
Magnetic wheel adsorption test. (**a**) Horizontal direction; (**b**) vertical downward direction; (**c**) vertical upward direction.

Based on the above analysis, the magnetic wheel with the same polarity arrangement has a large adsorption force that is positively correlated with number of magnets, thickness of yoke, and thickness of its hole shoulder. Combined with the above characteristic parameters and wheel weight considerations, the lightweight design of the magnetic wheel is carried out. Finally, a magnetic wheel is obtained with the following parameters as shown in Table 1:

We conduct experimental tests on 5-mm-thick flat steel plates and arc steel plates to measure the actual adsorption force of the magnetic wheel. As shown in the Figure 4, for different working conditions such as horizontal direction, vertical upward direction, and vertical downward direction, we use a force meter to measure the adsorption force by hanging a weight or pulling the wheel until it detaches from the steel board surface.

We make multiple measurements and record the data, as shown in Table 2:

Measurement data show that for the same surface, the magnetic wheel can provide a constant adsorption force for different working conditions. Moreover, for different wall surfaces, the magnetic wheel suction force is slightly affected by the contact state of the yoke and the surface. The greater the radius of curvature of the wall surface is, the better the contact between the magnetic wheel and the wall surface, the higher the magnetic conduction efficiency, and the greater the adsorption force. Based on the analysis of the above data, combined with the safety and reliability of adsorption, the developed magnetic wheel has a suction force of 220 N, and a mass of 700 g (7 N). The self-weight ratio of the suction force is as high as 31 (220 N/7 N), which is better than similar conventional magnetic wheels.

### 2.2. Passive Adaptive Flexible Detection Mechanism

The robot needs to closely fit the yoke to the surface to ensure the accuracy of the detection results during magnetic particle testing on the facade. Lifting force is measured to characterize magnetic field strength during excitation and ensure that magnetic field strength meets the need of the operation. Given that the robot works on a variable curvature facade, the yoke adaptively fits the variable curvature facade, and the lifting force’s accurate detection during the excitation process plays an important role to ensure the detection results and improve the detection accuracy. How to realize the flexible adaptation of multi-degree of freedom (DOF) of the detection mechanism and the accurate measurement of lifting force under close contact of magnetic yoke and wall (without relative motion) is a great difficulty. Therefore, this paper proposes a serial-parallel hybrid flexible adaptive mechanism to solve the problem of flexible adaptation of the curved surface. On this basis, a precision measurement unit is integrated to solve the problem of accurate measurement of the lifting force of the excitation, thereby ensuring that the requirements of high-precision flow detection under different walls are met. Finally, a passive adaptive compliance detection mechanism based on flexible deformation and high-precision detection is obtained.

**Figure 5:**
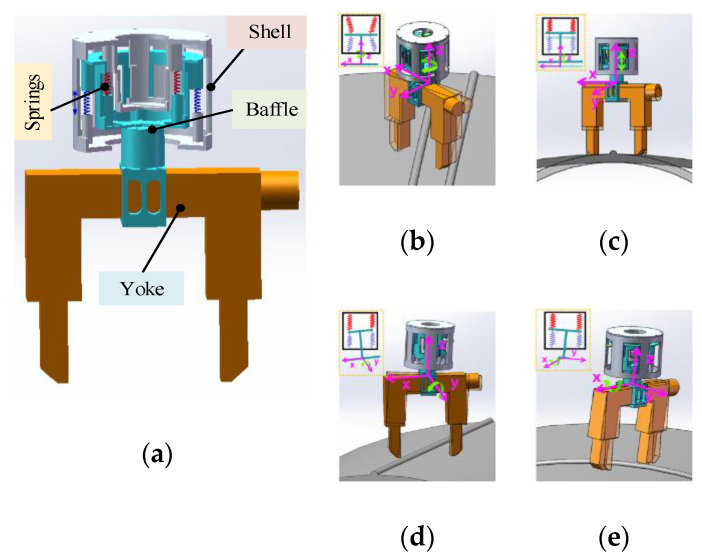
Flexible mechanism. (**a**) Structure of flexible mechanism; (**b**–**e**) principle of flexible adaptation.

The flexible mechanism consists of a shell, a baffle, and two groups of eight springs, as shown in Figure 5a. The component structure is optimized by using radial dislocation principle to make full use of space. The upper and lower cylindrical shells are rigidly connected to form a flexible mechanism shell, and two axially fixed series spring groups are inside. Each group consists of four parallel springs arranged in the circumferential direction, and the two ends of the spring group in the compressed state are fixed to the shell and the intermediate partition for connecting the yoke. The structure allows the intermediate baffle to move inside the housing flexibly, and finally obtains a flexible mechanism. Considering the gravity of the magnetic yoke, external forces on the magnetic yoke during the adaptive process, and the lifting force, we optimized the stiffness and length of the two sets of springs appropriately. In the process of the whole flexible mechanism driving the magnetic yoke close to the wall, the two groups of springs have different degrees of deformation, under the action of the magnetic yoke and the external force on the wall, which realizes the flexible adaptive mechanism. When the two-series spring groups expand and contract in the Z-axis direction, the yoke is driven to move along the Z-axis through the intermediate partition, which realizes the flexible adaptation of the Z-axis movement of the yoke. When the two-series spring groups undergo a circumferential torsional deformation around the Z-axis, the magnetic yoke is driven to twist around the Z-axis through the intermediate partition, which realizes the flexible adaptation of the yoke’s Z-axis rotation. When the two-springs in the X-axis direction produce different deformation variables, the intermediate baffle connected to the yoke tilts around the Y-axis, which realizes the flexible adaptation of the yoke to the Y-axis. When the two-springs in the Y-axis direction produce different deformation variables, the inclination of the connected yoke around the X-axis realizes the flexible adaptation of the rotation of the yoke along the X-axis. In summary, the passive adaptation of the four DOFs of the flexible mechanism realizes the yoke to fit the different curved surfaces.

**Figure 6:**
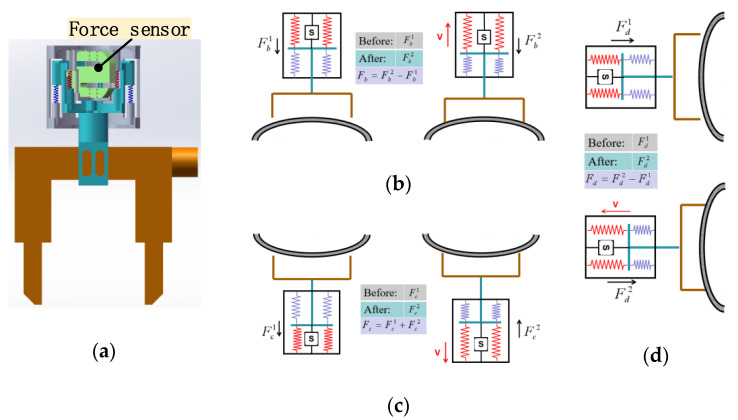
Flexible measurement method. (**a**) Force sensor; (**b**–**d**) measurement fundamentals.

A force sensor is fused in the flexible mechanism to achieve an accurate measurement of lifting force. Passive adaptation of the flexible mechanism and precise adjustment of force is used for the accurate measurement of the lifting force without a slight displacement between the yoke and the wall. In lifting force measurement, we first record initial force $F_{1}$ received by the force sensor under the reset condition. During excitation lift, we record pulling force $F_{2}$ of the force sensor affected by adsorption force; $F_{2}$ contains lifting force F and initial force $F_{1}$. F of the yoke is obtained by calculating the difference between $F_{1}$ and $F_{2}$. When F reaches the standard value, magnetic field strength during the excitation process meets the requirements of detection operation, stops the excitation operation, and restores the original position to complete the lifting force measurement. For different working conditions, the specific working principle of lifting force measurement is shown in Figure 6:

As shown in Figure 6a, the force sensor is integrated into the flexible mechanism, and force perception and measurement are realized through the magnetic yoke and its connectors. A schematic diagram of the lifting force detection of the horizontal bottom surface of the flexible detection mechanism is shown in Figure 6b. In the reset state, the force sensor receives pulling force $F_{b}^{1}$, and the force sensor receives pulling force $F_{b}^{2}$ during yoke excitation lifting. The difference, $\Delta =F_{b}^{2}-F_{b}^{1}$, is the lifting force $F_{b}$ received by the yoke in this state. A schematic diagram of the horizontal top surface lifting force detection process of the flexible detection mechanism is shown in Figure 6c. In the reset state, the force sensor receives pressure $F_{c}^{1}$, and in the yoke excitation lifting, the force sensor receives pulling force $F_{c}^{2}$. The difference, $\Delta =F_{c}^{1}+F_{c}^{2}$, is the lifting force $F_{c}$ received by the yoke in this state. A schematic diagram of the lifting force detection process of the vertical elevation of the flexible detection mechanism is shown in Figure 6d. In the reset state, the force sensor receives pulling force $F_{d}^{1}$, and during the yoke excitation lifting, the force sensor receives pulling force $F_{d}^{2}$. The difference, $\Delta =F_{d}^{2}-F_{d}^{1}$, is the lifting force $F_{d}$ received by the yoke in this state. The problem of accurate detection of the lifting force under different operating conditions is solved through the flexible adaptation of the flexible mechanism and the precise control of the force. When lifting force reaches the standard value, the magnetic field strength of the yoke meets the testing requirements to ensure the accuracy of the testing results.

## 3. Mechanical Analysis

The robot’s weight and adsorption stability directly affect its motion performance and work safety. When the detection robot works continuously under different facades and different working conditions, its gravity field and magnetic field constantly change. Therefore, the traditional mechanical model encounters difficultly in realizing the mechanical calculation under the time-varying force field, which brings difficulties to the overall optimal design. In this paper, the mechanical model of robot system with time-varying coupling force field parameters is established, and the mechanical analysis of robot system under different working conditions is solved by changing the value of time-varying parameters. The robot system is optimized to improve comprehensive performance, through the establishment of constraints.

We propose a robot mechanical analysis method based on the time-varying force field to analyze the mechanical state of the detection robot under different working conditions. Considering that the magnetic field will change with the change of the wall surface, a systemic unified mechanical model that can express the force state of the robot under different working conditions is established. When the robot is continuously detecting the facade surface, the direction of gravity is constant downward, and the direction of the adsorption force changes with the working surface but is always perpendicular to the wall surface, which results in an angle between the magnetic field and the gravity field. The direction of the support force is opposite to the direction of the adsorption force, the direction of the friction force is perpendicular to the direction of the adsorption force, and the magnitude of the support force and the friction force are linearly related to the adsorption. Based on the above analysis, the variable of the angle between the magnetic field and the gravity field is proposed, and a unitive systemic mechanical model in different states is established to analyze the force state of the robot.

**Figure 7:**
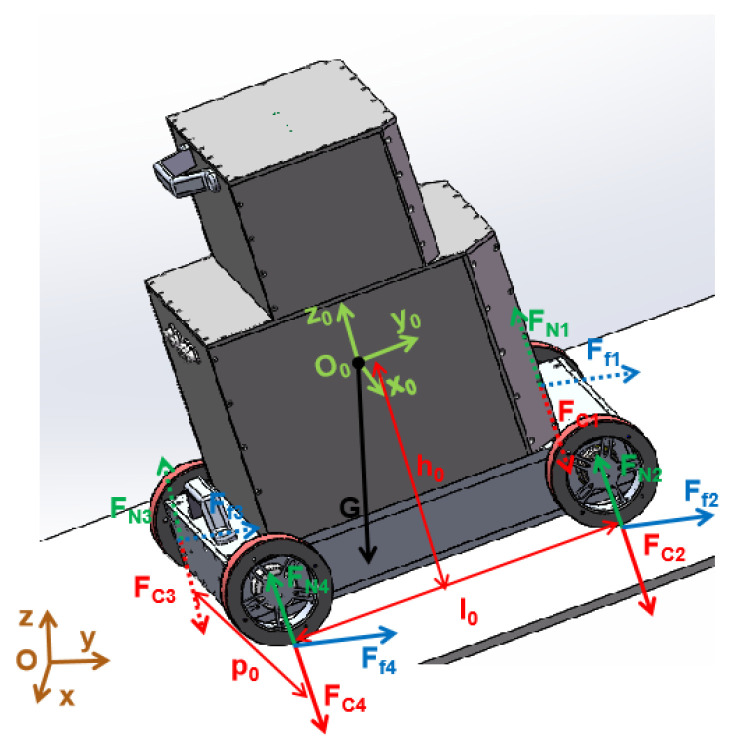
Mechanical model.

Based on the above method, the mechanical model of the detection robot system is established, as shown in Figure 7. The magnetic field angle between the magnetic field and the gravity field can be expressed by the angle of the robot gravity (G) direction in the robot body coordinate system ($O_{0}-x_{0}y_{0}z_{0}$), whose magnitude is [α β γ], and $\cos^{2}\alpha +\cos^{2}\beta +\cos^{2}\gamma =1$.

The mechanical model is based on the condition that all magnetic wheels are adsorbed on the wall surface. It is not aimed at the case where the magnetic wheel falls down from the wall affected by a large non-conductive obstacle, resulting a huge decrease in the adsorption force between the magnetic wheel and the wall. According to principles of static equilibrium and moment balance in classical mechanics, the robot mechanical model shown in the following formula is established:

(1) $$\{\begin{array}{c} \sum_{i=1}^{4}F_{fi}-G\sin\gamma =0\\ \sum_{i=1}^{4}F_{Ni}-\sum_{i=1}^{4}F_{ci}-G\cos\gamma =0\\ G\cos\beta \cdot h_{0}-G\cos\gamma \cdot \frac{l_{0}}{2}+(F_{c1}-F_{N1}+F_{c2}-F_{N2})l_{0}=0\\ G\cos\alpha \cdot h_{0}-G\cos\gamma \cdot \frac{p_{0}}{2}+(F_{c2}-F_{N2}+F_{c4}-F_{N4})p_{0}=0 \end{array}$$

The four equations in Formula (1) correspond to different states respectively: The first equation represents the equilibrium state in the plane $O_{0}-x_{0}y_{0}$; the second equation represents the equilibrium state in the $z_{0}$-axis direction; the third equation represents the moment balance state with overturning tendency along the axis of the two rear wheels; and the last equation represents the moment balance state with the rollover tendency. The meaning of the letters in the formula is shown in Table 3:

Since the adsorption force of the magnetic wheel is only related to its characteristics and wall thickness, we assume that the adsorption force of each magnetic wheel is the same to simplify the calculation, so $F_{Ci}=F_{C0}$. Through the equations of Formula (1), we can know that the static friction force is related to its gravity and the magnetic field angle; the supporting force is related to the adsorption force, robot’s structure parameters, and the magnetic field angle. Since the robot’s weight, and its structural parameters are known and the magnetic wheel’s adsorption force is fixed, the following equation can be obtained through simple derivation:

(2) $$\{\begin{array}{c} \sum_{i=0}^{4}F_{f}{}_{i}=G\sin\gamma \\ \sum_{i=0}^{4}F_{N}{}_{i}=4F_{C0}+G\cos\gamma \\ F_{N1}+F_{N2}=\frac{G(l_{0}\cos\gamma -2h_{0}\cos\beta )}{2l_{0}}-2F_{C0}\\ F_{N2}+F_{N4}=\frac{G(p_{0}\cos\gamma -2h_{0}\cos\alpha )}{2p_{0}}-2F_{C0} \end{array}$$

Considering that the robot must be safe to operate under various working conditions, the following are required: 1. The robot can safely absorbed on the wall without slipping, which requires that the maximum static friction force must satisfy the downward trend caused by gravity. 2. The robot can be adsorbed stably on the wall without falling, which requires a wall supporting force between the robot and the wall. 3. Robot can move steadily on the wall without overturning and rolling over, which requires a supporting force to keep the same side wheels from leaving the wall. Since the robot may work in any position in space, we can use the supporting force of the two front wheels (No. 1 and No. 2) and the two right wheels (No. 2 and No. 4) to represent the state of overturning and rolling over, respectively. In summary, the constraint equation to establish the safe motion of the robot is as follows:

(3) $$\{\begin{array}{c} \sum_{i=1}^{4}\mu_{1}F_{Ni}\geq G\sin\gamma \\ \sum_{i=1}^{4}F_{Ni}\geq 0\\ F_{N}{}_{1}+F_{N}{}_{2}\geq 0\\ F_{N}{}_{2}+F_{N}{}_{4}\geq 0 \end{array}$$

Through the above analysis, we can know that the motion performance and safety stability of the robot are closely related to its weight and magnetic wheel adsorption force. As the weight of the robot becomes smaller, its stable, accurate movement can be ensured because the resistance required to overcome the movement is reduced. As the adsorption force of the robot becomes larger, the higher the suction reliability when working on the facade, and the greater safety coefficient. Therefore, we need a lightweight robot with a suitable suction force. However, there is a contradiction between mass and adsorption force: as the weight of the robot decreases, adsorption force may decrease, which affects the safety of the operation. As the adsorption force increases, its weight may increase, which affects its motion performance. In order to get a wall-climbing robot with good performance, we need to carry out optimization design. Moreover, the size of the robot and the height of the center of mass effect the overall performance of the robot. Largely influencing the overall performance of the whole machine is difficult because the change range of the two is relatively small. Therefore, we mainly carry out an optimized design from the aspects of the robot’s mass and the wheel suction force to improve the performance of the robot comprehensively.

Combined with the mechanical model analysis and the above constraints, we can easily get that the weight has a great impact on the performance of the robot, so we first carry out the lightweight design. The weight of the robot is closely related to the robot’s external dimensions, the weight of the magnetic wheel, and the optimization method, whose parameters contains a large number of coupling factors, so it is difficult to establish the objective function similar to the traditional model. For this reason, we used multiple iterations to get the optimal value for the above parameters, and finally got the result of body mass of 30 kg and magnetic wheel adsorption force of 220 N. The structural parameters of the optimized detection robot, which has good sport performance, low weight, and high operational safety, are shown in Table 4:

## 4. Control System

The detection robot needs to realize multi-module operations through remote operation, such as precise movement, excitation detection, image acquisition, and fluorescent magnetic suspension spraying. The control system not only needs to meet the real-time and high-precision requirements of the control but also needs system integration and simplified processing. Moreover, it requires multi-component sensing and collaborative control to meet standard inspection. Under the requirement of stable transmission of interactive data by remote control, it is a key technical problem that we need to solve to realize high-precision synchronous coordination among multiple modules and actions. For this purpose, we independently control each module based on the RS485 bus to solve the real-time control of the multi-module cooperative operation. In this paper, multiple functions such as water, electricity, gas, and control are integrated and simplified through the functional division of the modules. Finally, a distributed bus controller architecture is formed to solve the high-precision cooperative control between multiple operating units in detection. Based on the in-depth analysis and research of the standardized detection process and combined with the structural characteristics of the robot multi-component and multi-sensor fusion, a flexible detection method based on multi-DOFs components and multi-sensor collaborative operation is proposed, which realizes the cooperative control of the highly integrated system of inspection robots and solves the problem of efficient, standardized detection operation.

The highly reliable integration of the hardware system and the real-time synchronization and precise collaborative control of multiple modules are the keys to realizing the remote operation of the detection robot and completing the flaw detection operation with the standard inspection. We integrate and simplify the motion module, detection module, lifting force measurement module, image acquisition module, fluorescent magnetic suspension spraying module, multi-sensor perception module, and other modules required for the detection. We deeply study the standard inspection and propose a multi-component sensing and collaborative control method. We use the bus mounting method to meet the real-time synchronization of the control of each module, achieve high-precision collaborative control between multiple operating units during inspection and complete the power supply, compressed air, magnetic suspension, Ethernet resource allocation management, and finally, inspection.

**Figure 8:**
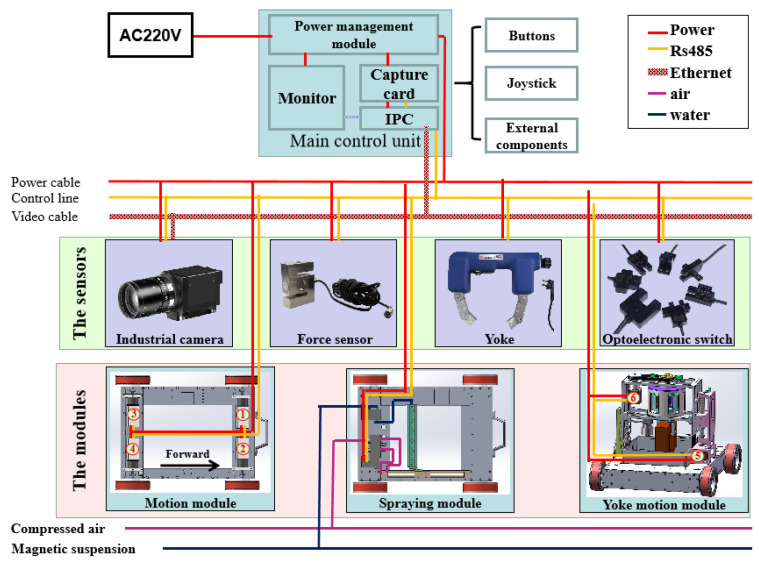
Control system composition.

As shown in Figure 8, the power supply and management of all equipment in the system are realized through the power management module in the main control unit; external sensing components on the main control unit, such as buttons, remote pole, keyboard, and mouse are used for human-machine interaction. RS485 bus and Ethernet are used for data transmission to solve the remote control and image transmission of the detection robot. We select 6 servo motors, whose serial numbers are shown in Figure 8, to drive the movement of the components for the detection process. And we can realize their real-time accurate control by sending speed information in real-time through the controller. Through the data transmitted by the RS485, motor numbers 1–4 in the motion module, which represent the four-wheel motors respectively, can be controlled accurately and synchronously to solve the high-precision motion problem of the robot. In the fluorescent magnetic suspension spraying module, the rodless cylinder and nozzle actions are controlled by RS485 bus to realize reciprocating spraying operation. The high-precision cooperative coordination between motor numbers 5 and 6, which are used to drive the yoke lifting and rotating, is achieved by RS485 to solve the problem of cross-excitation. The RS485 bus is connected to the detection module to control the on-off of the yoke relay, which solves the problem of excitation detection on the wall. We can accurately calculate the lifting force in different working conditions by reading the data of the force sensor. The optoelectronic switches are mounted on the vertical lifting mechanism and horizontal rotating mechanism, providing a position marker for its movement, and can be used as the limit switch to avoid mechanical damage caused by uncontrolled movement. An industrial camera is used to collect wall image information, and Ethernet transmits the image to the display of the main control unit quickly and stably in real-time. The actions of multi-module units are tightly and reasonably coordinated to achieve multi-line, multi-modular distributed collaborative control by constructing a multi-modular distributed bus architecture and combining human-like flexible operation technology, and the automatic detection operation is finally completed.

**Figure 9:**
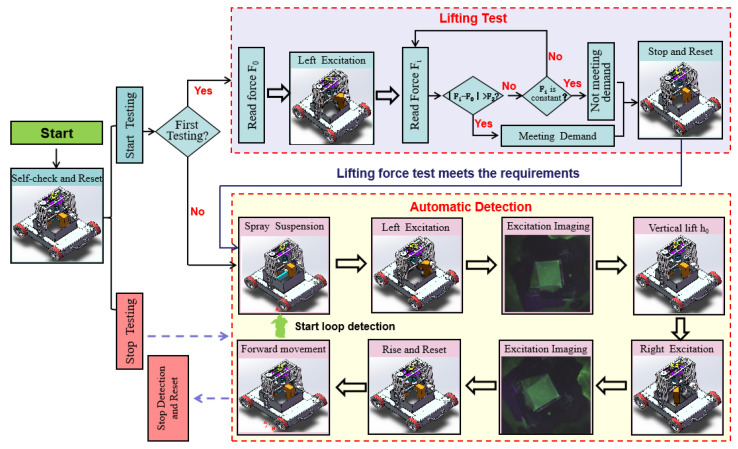
Automatic detection process.

Flexible operation and standard detection are important factors for promoting the robot’s efficient detection operation and meeting engineering applications. We analyze the human operation mode and divide the operation into units. The detection process is mainly divided into a robot motion control unit, a force measurement unit, a detection unit, and an image acquisition unit, as shown in Figure 9. Detection is composed of two parts: lifting force detection and automatic flaw detection. Lifting force test ensures that the magnetic field strength of the magnetic yoke meets the detection requirements, where the action (stop and reset) is the stop of lifting force test and mechanical reset of the magnetic yoke, rather than the entire detection process. The automatic detection operation completes the humanoid operation through the collaboration between multiple modules to achieve efficient inspection. Only when the lifting force test meets the requirements, will the robot system conduct the automatic detection process.

Reset refers to the mechanical reset of the magnetic yoke. The magnetic yoke rise and rotation are controlled through the coordination of motor numbers 5 and 6 to reach the zero position, and the reset is completed. The movement of the robot is completed by the moving mechanism with magnetic wheels. The robot can reach the initial position stably and accurately, and then perform lift force measurement and automatic detection work by adjusting the rotation speed and direction of motor numbers 1–4. The precise lifting force is obtained by using the calculation method mentioned in Figure 6 above, with the coordination of motor numbers 5 and 6, dynamometer, and magnetic yoke. We can know whether the magnetic field strength meets the detection requirements by comparing it with the standard lifting force. Once satisfying the conditions, the robot stops the lifting force test immediately and completes the mechanical reset of the magnetic yoke to prepare for the next step of the automatic detection process. If the conditions are not satisfied, the robot stops the lifting force test and completes the mechanical reset of magnetic yoke too, but the automatic detection process is temporarily terminated because of the insufficient magnetic field strength.

The process of automatic detection is as follows:

1. When the automatic detection instruction is received, the robot starts the Spray Suspension process. The solenoid valves of the rodless cylinder and nozzles are controlled to call the compressed air and the magnetic suspension, so as to realize the reciprocating spray of the magnetic suspension on the wall.
2. When the signal is detected again through the sensor at the initial position of the rodless cylinder, it indicates that the magnetic suspension spraying has been completed and then begins the Left Excitation process. The magnetic yoke can be controlled to rotate to left detection position, and descend to fit with the wall, through the coordination of motor numbers 5 and 6.
3. When a change in the dynamometer value is detected, that means the magnetic yoke touches the wall, the Excitation Imaging starts. The magnetic yoke starts to excite, and after 2 s, the camera begins to collect detection results. The image is transmitted to the main control unit by Ethernet and saved. Then the magnetic yoke stops exciting.
4. The magnetic yoke begins to lift after it stops working. Motor number 5 is precisely controlled to rise the magnetic yoke through RS485.
5. As the magnetic yoke rises to h0, the magnetic yoke Right Excitation is performed. The magnetic yoke can be controlled to rotate to the right detection position, and descend to fit with the wall again, through the coordination of motor numbers 5 and 6.
6. When a change in the dynamometer value is detected, the Excitation Imaging starts again. Energize excitation detection and take pictures, complete cross excitation detection, and record two wall detection images simultaneously.
7. The magnetic yoke begins to reset after it stops working. The magnetic yoke can be controlled to return to its zero position through the coordination of motor numbers 5 and 6.
8. When the magnetic yoke returns to its zero position, the vehicle body is controlled to advance to the next position by controlling motor numbers 1–4.

Then, repeat the above steps to complete the continuous autonomous detection operation until the stop signal is received. When the stop signal is received, the robot will complete the detection of the current position, and then terminate the entire detection process and complete the mechanical reset of the magnetic yoke.

## 5. Experiment

**Figure 10:**
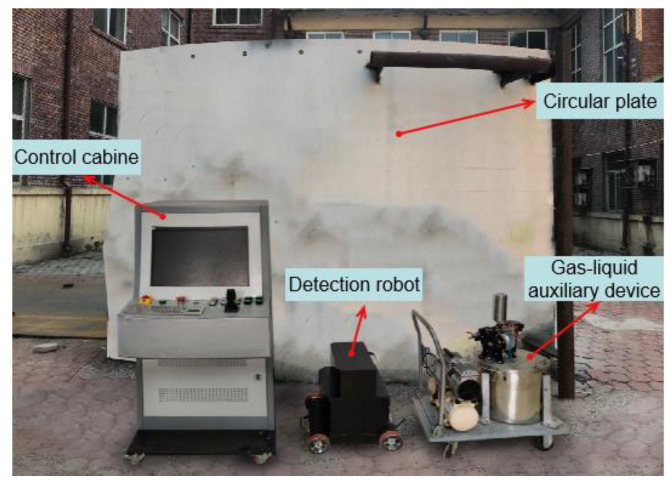
Experiment platform.

A series of performance tests are carried out to verify the reliability of the climbing robot system and the feasibility of the flexible detection method. The experimental environment is mainly composed of a detection robot system and a vertical arc facade, as shown in Figure 10. The detection robot system is mainly composed of a control cabinet, a flaw detection robot, and a gas-liquid auxiliary device. The robot is equipped with inspection equipment to complete the wall flaw detection through a series of detection actions. The control cabinet integrated by the control system hardware is used as the main control unit to carry out the inspection collaborative control of multiple components and sensors in the operation. The gas-liquid auxiliary device integrates an air compressor and a magnetic suspension diaphragm pump to provide compressed air and magnetic suspension and assist in the completion of a flaw. The operator can control various tasks of the robot through the control cabinet, and simultaneously monitors the status of the wall to be inspected in real-time; thus, the robot can perform automatic inspection operations instead of humans.

### 5.1. Motion Performance Test

As mentioned above, the detection robot needs to have adsorption stability and good motion performance because it works on the magnetically permeable facade. We conduct robot movement tests under different walls to verify its stability and accurate motion capabilities. As shown in the figure below, we perform motion tests on arc steel plates and arc plates with variable curvature and analyze their performance by observing the changes in the speed of the wheel motor, the position of the center of mass, and yaw angle of the robot. The wheel speed is obtained through the encoder, and the center of mass and yaw angle are obtained through inertial navigation.

**Figure 11:**
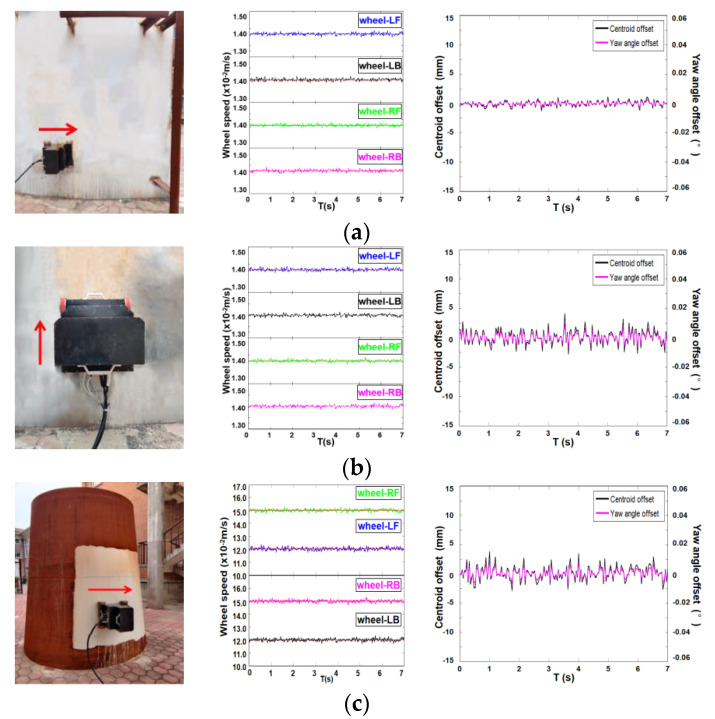
Sports performance test. (**a**) Horizontal circular motion on a circular steel plate; (**b**) vertical upward motion on a circular steel plate; (**c**) horizontal circular motion on a circular steel plate with variable curvature.

Figure 11a shows the detection of the upward movement of the robot arc steel wall: The speed of each wheel fluctuates slightly due to the efficiency of the transmission mechanism, but they all fluctuate around the speed of 0.14 m/s, and the position of the center of mass has a small offset in the vertical direction, within ±1 mm. Figure 11b shows the detection of the circular arc wall climbing motion of the robot’s arc steel plate: The speed of each wheel fluctuates around the speed of 0.14 m/s, and the position of the center of mass has a small offset in the vertical direction, within ±2.5 mm. Both cases indicate that the robot has a good stable motion performance on the circular steel plate. Figure 11c shows the detection of the horizontal climbing motion of the arc steel plate with variable curvature: Influenced by the radius of curvature of the wall, the rotation speed of the two left wheels is reduced to ensure the horizontal circular motion of the robot; the position of the center of mass has a small offset in the vertical direction, within ±5 mm, and almost no course deviation angle is generated. These above experimental results show that the designed moving mechanism based on permanent magnet wheels can meet the reliability and accuracy requirements of different wall motions and has a good motion performance.

### 5.2. Lifting Force Measurement Test

The inspection mechanism needs to have the ability of wall compliance and accurate measurement of lifting force to meet the magnetic field strength required for inspection and ensure the authenticity and reliability of the inspection effect when the inspection robot performs flaw detection on the wall surface with a variable curvature. As shown in the figure below, we conduct a lifting force test on the flat steel plate and the circular steel plate and observe the change in the force measurement value to verify the wall adaptation and accurate measurement capabilities of the passive adaptive compliance detection device.

**Figure 12:**
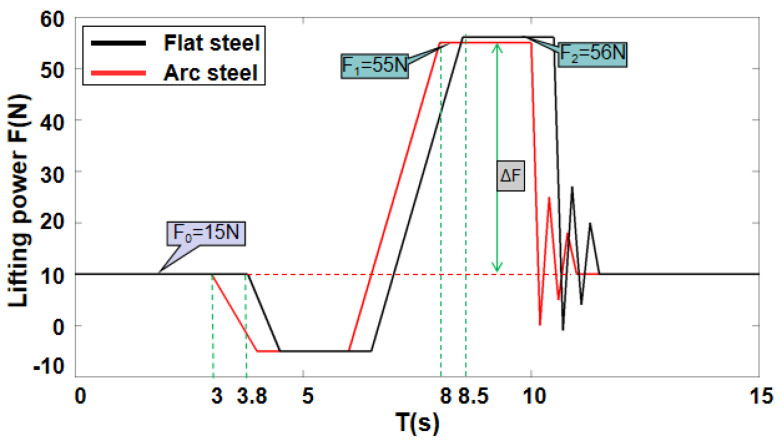
Flexible force test on two cases.

In Figure 12, the red curve shows the situation of the lifting force detection on the arc steel plate: The initial state force measurement value is $F_{0}$, and the yoke contacts the wall surface at time *t* = 3 s. After being completely close to the wall surface at time *t* = 4 s, the excitation lift starts, and the force measurement value gradually increases. At time *t* = 8 s, the lifting force $F_{a}=F_{1}-F_{0}$, meets the detection requirements, and then the yoke is powered off to complete the mechanical reset. The black curve shows the lifting force detection on the flat steel plate: The initial state force measurement value is $F_{0}$, which touches the wall surface at time *t* = 3.8 s. After being completely close to the wall surface at time *t* = 4.5 s, the excitation lift starts, and the force measurement value gradually increases. At time *t* = 8.5 s the lifting force $F_{b}=F_{2}-F_{0}$ meets the detection requirements, and then the yoke is powered off to complete the mechanical reset.

A comparison of the results of the two different wall lifting force measurement data shows that the yoke contacts the wall surface at different times in both states, but both can meet the wall surface closely, which means that the flexible adaptation mechanism solves the problem of flexible adaptation of the wall surface of the detection mechanism. The lifting force of the yoke is obtained by calculating the difference between the force sensor data before and after the excitation lift, which solves the accurate measurement of the lifting force without a slight displacement between the yoke and the wall. The above experimental results show that for different wall surfaces, the passive adaptive compliance detection mechanism designed can achieve wall surface flexibility adaptation and accurate measurement of lifting force.

### 5.3. Automatic Detection Experiment

**Figure 13:**
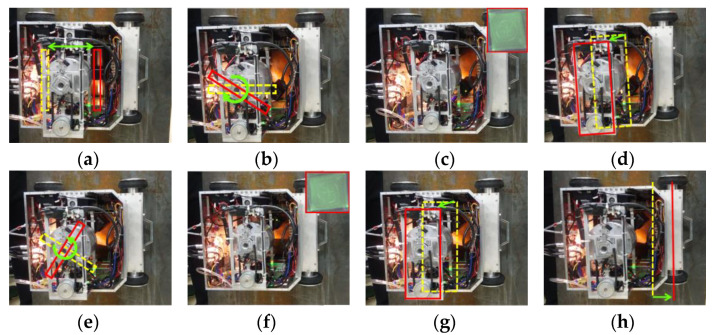
Automatic detection process. (**a**) Fluorescent magnetic suspension spraying; (**b**) the yoke turns right and goes down; (**c**) excitation and takes photos; (**d**) the yoke rises up; (**e**) the yoke turns left and goes down; (**f**) excitation and takes photos; (**g**) the yoke rises up to original height; (**h**) the robot goes ahead.

We carry out an automatic detection experiment to verify performance of the robot and the feasibility of the flexible detection method on the facade of the standard test piece with moderate sensitivity. The robot components can coordinate to complete the fluorescent magnetic particle testing and convey the test piece features by combining the multi-sensor information and the standard operation process. We can verify the detection capability of the robot system by observing whether the features of the test piece are clearly displayed. Figure 13 shows the typical movement during detection.

**Figure 14:**
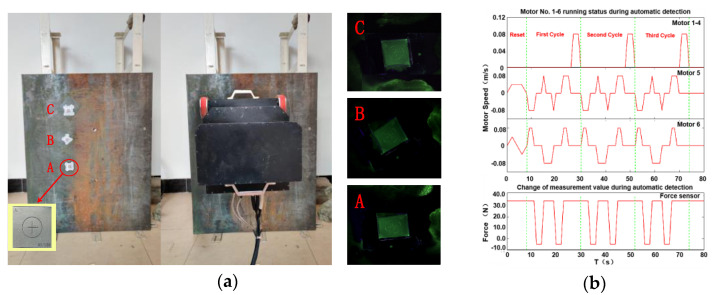
Continuous detection process. (**a**) Experimental platform and results; (**b**) changes of the robot motor speed and the force sensor.

We stick three test pieces on the facade surface for the continuous testing experiment and record the changes of robot motor speed and force sensor, as shown in the following figure:

Figure 14a shows automatic detection, including the wall to be detected, the test pieces, and the detection image collected. The three test pieces can be clearly displayed, indicating that the developed robot mechanism and testing process meet the testing requirements and realize efficient autonomous testing. Figure 14b is the time-varying curve of the movement of each driving component of the detection robot in the automatic detection operation. The figure shows that the automatic detection, whose cycle is 22 s, meets standard detection, and the robot’s detection workflows are closely and reasonably coordinated; the detection mechanism can adapt to the wall surface and then accurately measure and output the wall image through the camera to complete the task of fluorescent magnetic particle detection on the wall surface to be inspected. The above analysis confirms that the proposed automatic magnetic particle flaw detection is practical and has good periodicity and sustainability. The detection robot system can replace manual labor for efficient intelligent inspection.

## 6. Discussion and Conclusions

We develop a wall-climbing robot system for fluorescent magnetic particle testing to meet the increasing demand for automatic welding seam inspection in ships. Traditional wheel-type magnetic adsorption mechanisms mostly use circular magnets or permanent magnets placed at the bottom of the body, resulting in low effective utilization rate of the magnet, low magnetic wheel adsorption force, and an easy-to-scratch wall surface. In this paper, an innovative high-performance magnetic wheel is developed. The magnetic induction lines of array cylindrical magnets are collected by pure iron on both sides to avoid waste of magnetic energy and improve the utilization ratio of magnetic energy product. The moving mechanism adopts a four-wheel drive to meet the demand of high precision motion. During detection, the detection mechanism needs to adapt to the wall with different curvature and realize the magnetic yoke close to the wall to meet the magnetic field strength required by the detection. We propose a flexible mechanism based on the tandem and mixed connection, which solves the flexible adaptability problem of multiple DOFs through the flexible deformation of the springs. The force sensor is integrated to realize the accurate measurement of lifting force under the condition of no relative movement of the yoke and wall to ensure that the magnetic field strength meets the detection requirements.

Mechanical analysis must be performed in different working conditions to optimize the design of the robot. We put forward the concept of time-varying field parameters (the angle between gravity field and magnetic field), establish a unified mechanical model, and characterize the mechanical properties of the robot under different working conditions by changing time-varying parameters, thus completing the system optimization and improving the overall performance of the robot. Faced with the requirement of the robot’s multi-task, multi-component cooperation, high precision, and real-time remote control, we adopt bus mount control mode. According to standard detection, the robot system is divided into different modules, and different threads are established for control. Combined with sensor information, the coordinated control with high real-time and high synchronization is realized. This flexible detection method solves the close cooperation between the required magnetic suspension spraying, magnetic detection, image acquisition, and other operating processes, and realizes efficient detection.

The test results of the prototype test show that the developed fluorescent magnetic particle detection wall climbing robot system can achieve stable movement under the curvature of the magnetically conductive facade, its flexible detection device has good wall flexibility adaptation and accurate measurement of lifting force, and the cooperative control of multiple components and sensors realizes efficient detection. A method of combining multi-DOFs components and multiple sensors to formulate a multi-DOFs cooperative operation method is proposed, which realizes the cooperative control of the highly integrated system of inspection robots and solves the problem of efficient, standardized detection operation.

## Figures and Tables

**Table 1:** Magnetic wheel parameter table.

Item	Parameter
Dimensions	Φ120 mm × 35 mm
Wheel weight	700 g
Magnet parameters	20 × Φ10 × 30 mm
Magnet arrangement	Same polarity arrangement
Yoke thickness	2.5 mm
Hole shoulder thickness	2 mm

**Table 2:** Magnetic wheel adsorption force record table.

Wall Type	Times	Horizontal	Straight Down	Straight Up
Flat steel plate	First time	235 N	236 N	238 N
	Second time	233 N	231 N	234 N
	Third time	235 N	234 N	236 N
Arc steel plate (A) (12 m radius)	First time	230 N	229 N	228 N
	Second time	232 N	231 N	232 N
	Third time	235 N	232 N	236 N
Arc steel plate (B) (8 m radius)	First time	221 N	224 N	218 N
	Second time	219 N	223 N	221 N
	Third time	223 N	220 N	218 N

**Table 3:** Parameters in the mechanical model.

Symbol	Comment	Symbol	Comment
$O_{0}-x_{0}y_{0}z_{0}$	Robot body coordinate system	O−xyz	Natural coordinate system
$\mu_{1}$	Static friction coefficient	$p_{0}$	Width of detection robot
$l_{0}$	Length of detection robot	$h_{0}$	Height of the mass center
G	Gravity of the robot	i	Wheel number (1–4)
$F_{fi}$	Friction of each wheel	$F_{Ni}$	Wall support force of each wheel
$F_{Ci}$	Adsorption force of each wheel	$F_{C0}$	Average adsorption force of wheel

**Table 4:** System optimization results.

Item	Parameter	Item	Parameter
$l_{0}$	380 mm	G	30 kg
$p_{0}$	370 mm	$F_{Ci}$	220 N
